# Cellular Alterations Due to Direct and Indirect Interaction of Nanomaterials with Nucleic Acids

**DOI:** 10.3390/ijms25041983

**Published:** 2024-02-06

**Authors:** Miguel Encinas-Gimenez, Pilar Martin-Duque, Ana Martín-Pardillos

**Affiliations:** 1 Instituto de Nanociencia y Materiales de Aragón (INMA), CSIC-Universidad de Zaragoza, 50009 Zaragoza, Spain; miguelencinas@unizar.es (M.E.-G.); a.martin_pardillos@unizar.es (A.M.-P.); 2 Department of Chemical Engineering and Environmental Technology (IQTMA), University of Zaragoza, 50018 Zaragoza, Spain; 3 Ciber Bioingeniería y Biomateriales (CIBER-BBN), Instituto de Salud Carlos III, 28029 Madrid, Spain; 4 Departamento de Desarrollo de Medicamentos de Terapias Avanzadas (DDMTA), Centro de Terapias Avanzadas, Instituto de Salud Carlos lll, 28222 Madrid, Spain; 5 Instituto de Investigaciones Sanitarias de Aragón (IIS Aragón), 50009 Zaragoza, Spain

**Keywords:** DNA, nucleus, DNA damage, DNA breaks, ROS, nanoparticle, acid nucleic interactions

## Abstract

Deoxyribonucleic acid (DNA) represents the main reservoir of genetic information in the cells, which is why it is protected in the nucleus. Entry into the nucleus is, in general, difficult, as the nuclear membrane is a selective barrier to molecules longer than 40 kDa. However, in some cases, the size of certain nanoparticles (NPs) allows their internalization into the nucleus, thus causing a direct effect on the DNA structure. NPs can also induce indirect effects on DNA through reactive oxygen species (ROS) generation. In this context, nanomaterials are emerging as a disruptive tool for the development of novel therapies in a broad range of biomedical fields; although their effect on cell viability is commonly studied, further interactions with DNA or indirect alterations triggered by the internalization of these materials are not always clarified, since the small size of these materials makes them perfectly suitable for interaction with subcellular structures, such as the nucleus. In this context, and using as a reference the predicted interactions presented in a computational model, we describe and discuss the observed direct and indirect effects of the implicated nanomaterials on DNA.

## 1. Introduction

Nanomaterials have become, in recent years, an innovative alternative to solving existing problems, offering a wide range of properties that would not be available if working with average-sized materials. Paraphrasing Richard Feynman, when we enter the very small world (of atoms), many new things can happen, resulting in completely new opportunities for design [1].

Some unique properties of these materials, which generally range from 1 to 100 nm, include optical or fluorescence properties, high surface-to-volume ratio, or enhanced electrical conductivity [2]. Due to their special characteristics, nanomaterials are being used in a broad variety of fields, from the production of food additives, flavors or packaging with enhanced properties, to the adsorption of organic and inorganic pollutants for purification, or the development of improved devices for energy storage [3,4]. Particularly remarkable is the progress made in recent years in the field of biomedicine with the development of different nanomaterials for a wide range of applications: in the treatment of diseases, as a vehicle for the specific delivery of diverse pharmaceutical agents, or as key components of implants [5].

The rapid development of these peculiar materials in a great variety of fields, however, has led to the need for exhaustive biodistribution and toxicity studies of each specific nanomaterial [6]. Currently, the precise interactions between these material and cells are not completely understood; key factors such as the size, surface charge or specific functionalization are involved in the cytotoxic effects [7].

**Figure 1:**
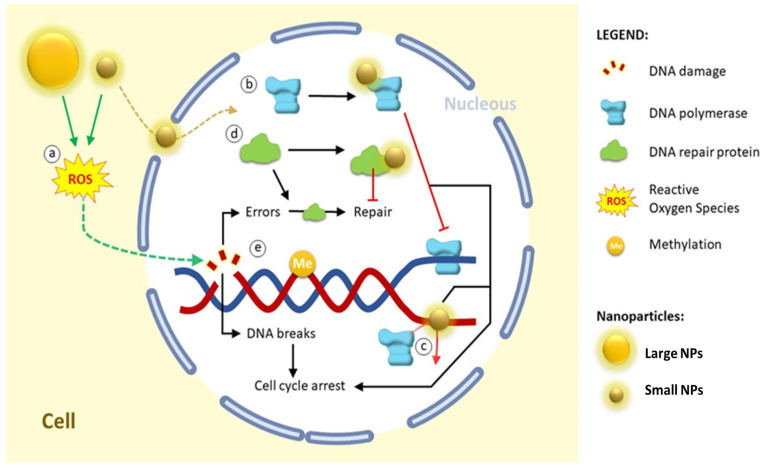
Effect of NPs with DNA. Internalization in the nucleus is size-dependent in general terms. (**a**) Large NPs cannot be internalized in the nucleus, although they can generate ROS and indirectly induce DNA damage. Small NPs can also generate ROS and can additionally be internalized and interact with nuclear proteins. (**b**) Interaction with DNA polymerase stops replication by inhibiting the interaction of DNA polymerase and DNA; and additionally, (**c**) NPs can bind to DNA and inhibit replication by blocking the binding of DNA polymerase and DNA. In cases where endogenous or exogenous DNA damage (NPs effect) is generated, this can be repaired by DNA damage repair proteins; however, (**d**) some NPs are able to interact with these repair proteins, blocking the repair process. These alterations lead to DNA damage accumulation and, as a consequence, cell cycle arrest; (**e**) additionally, several NPs can also modify the methylation profile (epigenetic alterations).

Concerns regarding the safety and health effects of nanoparticles (NPs) are related to possible interactions with essential cellular molecules, such as deoxyribonucleic acid (DNA) [8]. Nucleic acids are the principal informational molecules of the cell. DNA is defined as the genetic material of the cell, and different types of ribonucleic acid (RNA) participate in diverse cellular activities, including protein synthesis and functions as messengers [9]. Interaction with NPs could have an adverse effect (i.e., epigenetic alterations) on DNA (Figure 1) and its genetic functions, such as transcription, replication, and repair processes, which are crucial to maintain the normal metabolism of the living cell. Entry into the nucleus is, in general, somewhat difficult, as the nuclear membrane is a selective barrier to molecules longer than 40 kDa. Nevertheless, NPs are able to induce indirect effects through reactive oxygen species (ROS) generation [8]. However, in some cases, the size of the NPs can enable them to be internalized into the nucleus, thus exerting a direct effect on the DNA structure.

This review focuses on several commonly used nanomaterials, describing the most relevant reported direct and indirect interactions with DNA as well as observed genotoxic effects in living cells and organisms.

## 2. Computational Study of the Direct Interaction of NPs and DNA

**Figure 2:**
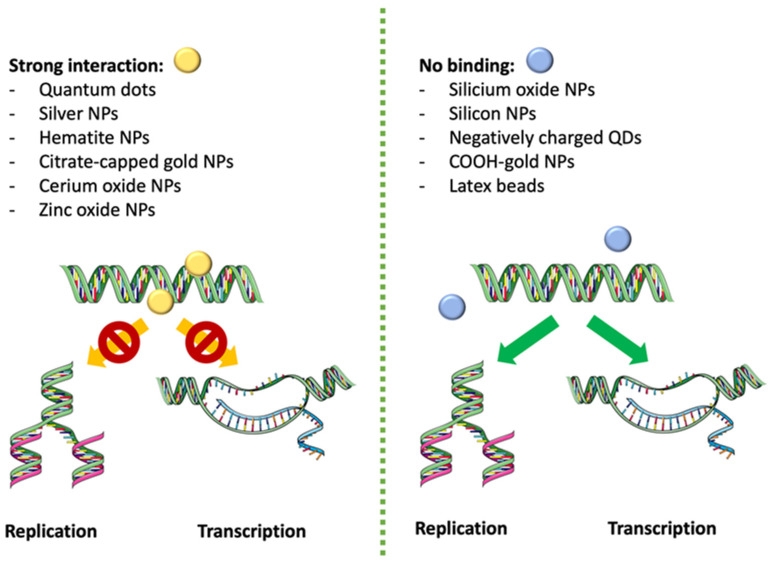
Effect of NPs on DNA functions according to interaction degree. Nanoparticles that strongly interact with DNA, as quantum dots (QDs), silver nanoparticles (Ag NPs), hematite NPs, citrate-capped gold nanoparticles (citrate Au NPs), cerium oxide nanoparticles (CeO_2_ NPs) and zinc oxide nanoparticles (ZnO NPs). They bind to DNA and inhibit the replication and transcription, whereas when there is no interaction (as for example with silicium oxide NPs, silicon NPs, negatively charged QDs, COOH-gold NPs and latex beads), replication and transcription are not altered.

Predictive models can represent a beneficial instrument for researchers to characterize physical interactions, e.g., the interaction of NPs with DNA molecules. A computational study has been able to predict the affinity of twelve types of NPs for DNA, concluding that NPs with a high affinity for DNA strongly inhibited DNA replication and transcription, whereas NPs with a low affinity had no or minimal effects on DNA replication. The model predicts how cationic quantum dots (QDs), silver nanoparticles (Ag NPs), hematite NPs, citrate-capped gold nanoparticles (citrate Au NPs), cerium oxide nanoparticles (CeO_2_ NPs) and zinc oxide nanoparticles (ZnO NPs) bind to DNA and inhibit in vitro replication (verified by quantification of polymerase chain reaction (PCR) products), whereas silicium oxide nanoparticles (SiO_2_ NPs), silicon NPs, negatively charged QDs, COOH-gold nanoparticles (COOH-Au NPs) and latex beads do not bind to DNA molecules [10] (Figure 2). Both behaviors were consistent with experimental data.

In this context, although titanium oxide nanoparticles (TiO_2_ NPs) are bound to DNA, no replication inhibition was observed. This phenomenon may be explained by the enhanced conductivity of TiO_2_ NPs, which could increase PCR efficiency. However, this is not the only plausible explanation, since it has been shown that TiO_2_ NPs inhibit DNA replication in a dose-dependent manner [11,12]. Other studies have described the genotoxicity induced by TiO_2_ NPs through oxidative stress generation [13]. The disparities observed among NPs-PCR studies have also been noted with the claim that the use of different PCR systems, polymerases and DNA templates leads to conflicting conclusions. Therefore, a standardization of the experimental conditions is needed in order to compare results between studies [12].

Despite the possible limitations of the theoretical model proposed by Li et al. [10], and the differences in the conclusions drawn compared with in vitro studies, it has also been reported that several of the above-cited nanomaterials have, due either to direct or indirect interactions with DNA, a genotoxic effect on cells.

## 3. Effects of NPs on DNA: In Vitro and In Vivo Studies

As it has been previously mentioned, despite the broad variety of implementations that nanomaterials are bringing to diverse areas of biomedicine, undesired behaviors related to cellular entities need to be thoroughly assessed. These events can lead to an important toxicity of healthy cells, which implies a concerning phenomenon, especially when the nanomaterial is administered to a living organism. The existence and characteristics of those side effects will determine the success of the nanomaterial as a therapeutic agent. However, the prediction of those effects usually produces quite complicated results.

Firstly, the potential risk associated with nanomaterials is not only limited to the interaction with cells but also to possible changes that the medium provokes in the nanoparticle or a potential degradation of the latter [14]. In this context, as it is described subsequently, ROS production and DNA damage result in a relevant toxicity mechanism at a cellular level alongside protein modification and the disruption of membrane integrity. Some of them were suggested to trigger several kinds of epigenetic alterations [15]. Likewise, it is possible that corrosive tissues’ microenvironments and lysosomes degrade nanoparticles, liberating potentially dangerous metal ions [16].

Secondly, the administration or exposition of the NPs is also an important factor, since they will likely interact with a certain organ depending on the method of entrance. Once they are inside the organism, they become covered by the protein corona (which can obviously alter its original function), and they can either interact with initially encountered organs and tissues or either translocate to the bloodstream and accumulate in distant organs [15,16]. In any case, they can potentially cause toxicity where they accumulate. For example, airborne nanoparticles may arise due to NPs deposition in lung tissues, leading to oxidative stress-mediated lung inflammation. Similarly, organs dedicated to elimination and excretion (i.e., liver, spleen, kidneys) are involved in the processing of NPs of different sizes [16]. Thus, abundant exposure to this material can potentially lead to an accumulation and ulterior severe effects. Nanoparticles have also been demonstrated to cross the blood–testicle and blood–placenta barrier, and some IONPs were associated with myocardial damage due to iron accumulation as well.

In addition to these issues, the physicochemical properties of the nanomaterial itself have a big influence on the potential side effects. It is crucial to perform a full characterization of the NPs since, as it has been widely reported, these properties strongly influence the cytotoxic capacity of the material, which also will be dependent on its interactions with the biological environment [16].

Fortunately, increasing research in nanomaterials during last years has provided some insight into the general behavior and considerations about NPs toxicity. In this context, size might be one of the most relevant properties that will determine the cytotoxic effect of the material. Related to this, researchers generally observed an inverse correlation between the size of the NPs and their toxicity either in vivo and in vitro [17]. As an example, the research group of Pan observed that 1.4 nm Au NPs exhibited a high in vitro toxicity in contrast to the non-observed toxicity of 15 nm Au NPs [18]. A similar effect was observed with silver NPs [19]. Another study showed that size variations in Au NPs could even determine the preferential site of in vivo accumulation [20].

Alongside this, it is also relevant to consider possible aggregations of the material, which are usually caused by the interactions with biological media previously mentioned. Several works have demonstrated that agglomeration can notably change NPs properties and toxicity, for example in ZnO NPs [21] or Au NPs [22]. As this phenomenon seems to be unavoidable when performing in vivo studies, the thorough study of potential aggregates in therapeutic nanomaterials results crucial [22]. In the same manner, other properties are equally important for notable cytotoxic variations and need to be thoroughly assessed for every material, such as shape or surface chemistry [16].

Nanomaterial-based therapies have experimented an exponential growth during last years. However, some aspects such as the above-mentioned have made the development of these materials difficult to a great extent. As a result, it is extremely complicated to predict the behavior of these materials in vivo in the face of some critical phenomena such as the protein corona formation, the aggregation, or in general, the fact that due to the interactions with the biological medium, a slight variation in the properties or composition of the nanoparticles leads to a notorious change in the capacities of the material.

To this end, researchers are constantly seeking new strategies to avoid this compositional changes or agglomeration. Among them, the decoration of NPs with some organic molecules results in a broadly used strategy, for example, using silica coating and polymer encapsulation to control ion release from NPs in order to mitigate ROS production [16].

Among all these strategies, the polyethylene glycol (PEG) decoration of NPs has been one of the most used, since it decreases the phagocytic uptake and reduces accumulation in non-target organs [17], also showing slower degradation and clearance rates for some NPs [23]. This coating has been even used for reducing NPs toxicity in the clinic [15]. However, recent times have raised concerns about this coating, which are mainly due to the development of antibodies and immune response mediated by anti-PEG antibodies in humans [15,16]. All these facts have led to the focus on the development of biocompatible, biodegradable and non-toxic nanomaterials. Under this premise, polymeric and organic nanomaterials are gaining importance, as well as some biodegradable synthetic polymers, such as poly(lactic acid) (PLA), poly(glycolic acid) (PGA), and poly(lactic-co-glycolide), which have been approved for the FDA and EMA [14]. Altogether, this progress will help overcome the drawback that some nanoparticles currently present related with their toxicity and undesired biological interactions, allowing higher specificity and efficiency.

This detailed background confirms that depending on some physicochemical properties, cellular effects caused by a nanomaterial can dramatically change. Thus, herein, we present a revision of nanomaterials differing in several features (material, size, decoration etc.), and their diverse effects on DNA through direct and/or indirect interactions.

### 3.1. Silver Nanoparticles (Ag NPs)

Ag NPs are able to induce DNA alterations through direct interaction with DNA and also through indirect effects such as increased oxidative stress, which can lead to cellular mechanism disruptions. In this context, starch-coated Ag NPs can enter the mitochondria and the nucleus in normal human lung fibroblast cells (IMR-90) and human glioblastoma cells (U251-MG), generating mitochondrial toxicity and DNA damage in a dose-dependent manner. The presence of Ag NPs in the mitochondria disrupts the mitochondrial respiratory chain, leading to the production of ROS and interruption of the ATP synthesis, which induces DNA damage. This damage is augmented by internalization of the Ag NPs in the nucleus, where the interaction of Ag NPs to the DNA leads to cell cycle arrest in the G2/M phase [24]. ROS generation is closely related with DNA damage induction [25], and it has been reported by other authors after Ag NPs treatment: in hepatoma cell lines with 5 nm Ag NPs [26], in a mouse hippocampal neuronal cell line (HT22) with commercial 20 nm Ag NPs [27], or in human bronchial epithelial BEAS 2B cells with Ag NPs between 43 and 260 nm in size [28].

DNA-strand breaks induced by Ag NPs have been also detected in human testicular cell line Ntera2 in a size- and dose-dependent manner [29]. A possible mechanism underlying Ag NP genotoxicity was reported by Butler et al., since they observed that DNA- strand breaks and micronuclei formation in Jurkat Clone E6-1 and THP-1 cell lines after Ag NP treatment was inversely correlated with the size of the NPs. This phenomenon can be explained by the greater release of Ag ions as the size of the Ag NPs decreased [30]. Nevertheless, some crucial aspects of the NPs, such as the coating, may result in meaningful changes in their effect. For instance, polyvinylpyrrolidone (PVP)-coated 5 nm Ag NPs caused oxidative stress in TK6 cells by themselves and without ion release mediation [31]. PVP-coated 42.5 nm Ag NPs, however, showed negative results for micronuclei formation or chromosomal aberration induction in human bronchial epithelial BEAS 2B cells, which was attributed to the efficient cell repair of DNA damage [32].

Also important is the assessment of the behavior of nanomaterials in vivo, since the effect of NPs can dramatically change when they are introduced into a complex organism. In this context, the induction of ROS-dependent DNA damage by Ag NPs has also been confirmed in vivo in a B6C3F1 mice strain after treatment with PVP- and silica-coated Ag NPs of different sizes. However, some differences were found depending on the coating, since PVP-coated Ag NPs caused toxicity on the bone marrow, whereas this phenomenon was not observed after treatment with silicon-coated Ag NPs [33]. Treatment with Ag NPs ranging in size from 6.3 to 629 nm also induced an alteration in bone marrow in Sprague–Dawley rats, including aberrated and polyploid cells. Additionally, histopathological changes in several organs such as the liver, kidneys or spleen were observed [34]. Bioaccumulation in the liver, and in the kidney, has been also corroborated after the oral administration of Ag NPs [35,36]. The presence of Ag bound to high-molecular proteins has also been demonstrated, revealing the presence of Ag(I) released by the oxidation of Ag NPs in the biological environment [36].

Positive results of genotoxicity have been obtained in both in vivo and in vitro assays. Rodriguez-Garraus et al., however, in a very complete and detailed review on the genotoxicity of silver nanoparticles, concluded that the characteristics of Ag NPs and test conditions should be considered case by case, since none of the 43 studies that they collected included a complete battery of experiments, as recommended by the International Council for Harmonisation of Technical Requirements for Pharmaceuticals for Human Use (ICH) and European Food Safety Authority (EFSA) guidelines [37].

A summary of the effects generated by Ag NPs is presented in Table 1.

### 3.2. Cerium Oxide Nanoparticles (CeO_2_ NPs/Nanoceria)

Currently, cerium oxide (CeO_2_) is being widely used in a variety of applications such as television tubes, glass/ceramic polishing agents, fuel cells, solar cells, gas sensors and ultraviolet absorbents [38]. In particular, CeO_2_ nanoparticles (also known as ‘nanoceria’) are released from diesel engines that use cerium compounds as a catalytic agent to decrease the diesel exhaust particles [39]. Considering this human inhalation exposure, it is important to identify the potential risks of this nanomaterial.

Although there is a wide range of works that report on the ability of CeO_2_ NPs to bind or adsorb DNA molecules [40,41,42], no study has focused on an in situ genotoxic effect due to the binding of pure CeO_2_ NPs with DNA in live mammalian cells. At best, the most suitable work that might confirm this feature is the study performed by Link et al., in which they show that CeO_2_, among other metallic NPs, successfully transfected several mammalian cell lines [43]. Several effects on DNA have been described, although the potential risks or benefits of CeO_2_ nanomaterials remain unclear. All these differences between studies may be related to the concentration, cell lines and animal models and also to the technique employed. For instance, some studies have reported that the Comet assay can be misleading due to the fact that CeO_2_ NPs interfere with the measurements [44,45].

#### 3.2.1. CeO_2_ NPs Cause DNA Damage

It has been widely described that CeO_2_ nanoparticles have a potential genotoxic effect, causing DNA damage. In this context, CeO_2_ NPs with a size range between 8 and 20 nm were able to generate ROS, diminish glutathione concentration, and generate nuclear fragmentation, chromatin condensation and apoptotic bodies in A549 cells. Increased caspase-3 and caspase-9 expression was also observed, as well as p53 phosphorylation and PARP-1 cleavage, thus concluding that ROS-mediated DNA damage plays an important role in CeO_2_-induced apoptotic cell death [46]. The generation of intracellular ROS has also been confirmed in A375 cells after treatment with 38 nm CeO_2_ NPs as well as glutathione (GSH) depletion, chromatin condensation, DNA strand breakage and increased caspase-3 activity [47]. Smaller CeO_2_ NPs (7 nm) have also been shown to induce DNA damage in human spermatozoa [48] as well as micronuclei formation and an intracellular GSH/oxidized glutathione (GSSG) ratio decrease, which is related to the induction of lipid peroxidation in human dermal fibroblasts. This latter phenomenon has been related to a clastogenic mechanism of chromosomal damage, leading to structural chromosomal aberrations [49].

Likewise, the study of the potential cytotoxic effects of these nanomaterials in living organisms is crucial. As described above, the most common way of interacting with CeO_2_ nanoparticles is inhalation. In this respect, A. Nemmar has studied in depth the effects of nanoceria in several animal models after the inhalation of this nanomaterial. Firstly, they studied the effect of commercial 20 nm CeO_2_ NPs after acute oral administration in BALB/C mice (0.5 mg/kg). It was observed that ROS levels significantly increased in the lung, heart, kidney and brain, and most importantly, the Comet assay showed DNA damage in all the organs studied (lung, heart, liver, kidney, spleen and brain) after treatment with CeO_2_ NPs [50]. They also evaluated the response of a rat model (male Wistar rats) of acute kidney injury (AKI) to the oral administration of 20 nm CeO_2_ NPs (1 mg/kg). Even though AKI-induced rats showed an increase in DNA damage compared to control rats, the Comet assay also revealed that this damage was significantly exacerbated when AKI rats were treated with CeO_2_ NPs, thus revealing that the inhalation of this nanomaterial may have more serious effects in individuals with renal diseases [39,51]. Similarly, they demonstrated the DNA damage caused by these nanoparticles in the aortic tissue in a vascular damage model in Wistar rats [52]. Related with these studies, in a very complete work, Kumari et al. described the effects of repeated oral administration of <25 nm CeO_2_ NPs or microparticles (MPs) during 28 days in Wistar rats. The doses chosen were higher than in the other studies (30, 300 and 600 mg/kg), and they observed a significant increase in DNA damage in liver and peripheral blood leukocytes, micronuclei and chromosomal aberrations in bone marrow, and micronuclei in peripheral blood after the treatment with CeO_2_ NPs at 300 and 600 mg/kg bw/day [53].

#### 3.2.2. CeO_2_ NPs Protect from DNA Damage

A wide variety of works have described a positive effect of these nanomaterials in cells when treated at low (generally non-cytotoxic) concentrations, which is noteworthy. A relief of oxidative stress and reduction in DNA damage caused by potassium bromate (KBrO_3_), a well-known oxidative stress-inducing agent, at subcytotoxic concentrations (from 2.5 to 7.5 µg/mL) has been described in the BEAS-2B cell line [54]. Comparable results were obtained with similar 25 nm CeO_2_ NPs in male albino Wistar rats. Pre-treatment of four intraperitoneal injections of CeO_2_ NPs at 0.01 µg/kg alleviated DNA fragmentation in the liver caused by D-galactoseamine and lipopolysaccharide (D-GALN/LPS) in a male albino Westar rat [55]. Additionally, it was shown that CeO_2_ NPs are not able to produce either genotoxicity or stress oxidative damage in Caco-2 cells [56].

Another toxicity-related factor is the shape and size of the nanoparticles, these being associated with different effects. A detailed study of several CeO_2_ nanomaterials in HepG2 cells concluded that octahedron-like (10–30 nm) and rod-like (8 nm diameter, 100–400 nm length) nano-CeO_2_ protect and inhibit DNA damage by scavenging (hydroxyl radical, ·OH), whereas 20–50 nm cube-like nano-CeO_2_ did not present this property [57]. Of note is a recent work which, in contrast to the other in vivo studies cited above, shows that the oral administration of 0.5 mg/kg of <25 nm CeO_2_ NPs in Swiss albino mice ameliorated the genotoxicity induced by lead acetate, significantly reducing DNA damage. The authors attribute these results to the possible antioxidative capacity of CeO_2_-NPs given its ability to switch reversibly between the III and IV oxidation states [58]. Another study also highlights the capacity of small polymer-coated nanoceria to diminish DNA oxidation caused by glutamate [59].

In addition, several studies have described the ability of nanoceria to protect against several kinds of irradiation. It has been reported that 10 nm CeO_2_ NPs prevent DNA damage and ROS production after ultraviolet (UV)-A irradiation and UV-B micronuclei formation in Jurkat cells [60]. Other studies have shown that pre-treatment with CeO_2_ NPs can significantly reduce DNA damage caused by X-ray irradiation in human bone marrow-derived mesenchymal stromal cells [61] and in MC3T3-E1 osteoblast-like cells [62] at 7 and 6 Gy, respectively. Protection against DNA damage has also been confirmed in vivo, where small CeO_2_ NPs were able to protect sperm from DNA damage after X-ray irradiation up to 5 Gy in C57BL/6J male mice [63].

A summary of the effects generated by CeO_2_ NPs is presented in Table 2.

### 3.3. Gold Nanoparticles (Au NPs)

Among all kinds of metallic nanoparticles, gold nanoparticles play a key role in the field of nanotechnology due to their size- and shape-dependent physicochemical and optoelectronic properties. Also noteworthy is the important role that the functionalization of surface-modified Au NPs play in several applications, such as biosensors, biodiagnostics or DNA and drug delivery. These properties postulate Au NPs as holding great promise in industrial and advanced medical applications [64].

#### 3.3.1. Au NPs Induce DNA Damage

It is well known that surface modifications or coatings can strongly influence the effects and behavior of nanomaterials in the cellular environment. Regarding Au NPs, the stabilization and reduction in chloroauric acid (HAuCl) with citrate salts, resulting in citrate Au NPs, is one of the most common syntheses in the field of nanomaterials. Through the use of predictive models, citrate Au NPs have shown a strong affinity for DNA with the expectation of a significant inhibition of the replication [10]. The ability of this material to adsorb DNA has been reported in vitro, in relation with single-stranded DNA (ssDNA) [65], and double-stranded DNA (dsDNA) [66], although it seems that ssDNA is favored for selectivity over dsDNA adsorption [67].

Several works have described a potential genotoxic effect of citrate Au NPs which leads to DNA damage and seems to be related with the dose and coating. In particular, HepG2 cells have shown significant DNA alterations after treatment with this kind of nanomaterial in diverse studies. In this context, 18 nm citrate Au NPs are able to cause DNA damage to these cells, which is accompanied by a significant reduction in viability [68]. Similarly, an increase in DNA damage in the same cells upon treatment, also with 18 nm citrate Au NPs, was observed, which did not take place when Au NPs were coated with 11-mercaptoundecanoic acid. In these cases, neither showed a significant reduction in viability for concentrations below 200 µM [69]. Furthermore, a complete study compared the behavior of Au NPs coated with different ligands in HepG2 cells, revealing that Au citrate NPs (~18 nm) were non-toxic overall, although they caused DNA damage that the cell could not repair [70], which may be related with the reduction in ATP production observed when these cells are treated with citrate Au NPs [71].

Other studies have reported a genotoxic effect of this kind of nanoparticles in other cell lines. In this context, 14 nm and 20 nm citrate Au NPs showed a significant increase in micronuclei and nucleoplasmic bridge production in CaCo-2 and HaCaT cells at 5 nM concentration [64]. Related to this, a study on a Chinese hamster ovary (CHO) cell line was conducted with 14 nm and 20 nm citrate Au NPs. It was determined, on the one hand, that due to the interferences of Au NPs with nucleated DNA, the Comet assay was not suitable for genotoxicity assessment of nanomaterials, as reported in a similar way by the works cited above. On the other hand, it has also been shown that citrate Au NPs may have some genotoxic potential in mammalian cells at the tested concentrations (6.25–50 µg/mL); however, the genotoxicity observed, although it did exist, was not statistically significant [72].

It seems therefore that the differences in genotoxic effects can be explained through the different mechanistic pathways involved in each nanomaterial according to its size and concentration. In this context, 30 nm citrate Au NPs have been related with the in vitro induction of genotoxicity, the authors stating that the genotoxicity of Au NPs with sizes greater than 20 nm had been rarely reported [73]; together with 10 nm citrate Au NPs, the same size Au NPs have also been reported to cause DNA damage in several structures (i.e., liver, blood and brain) in vivo [74,75].

It is also worth noting that citrate has been shown to have a cytotoxic effect by itself, causing apoptosis in two different gastric cancer cell lines [76]. It has also been suggested that an excess of sodium citrate, rather than NP size, may affect the viability of A549 and NCIH441 compared to dialyzed and purified Au NPs [77]. Similar observations have also been noted for HepG2 cells [70].

A summary of the effects generated by Au NPs is given in Table 3.

#### 3.3.2. Nuclear Internalization of Au NPs

In a living cell, nanoparticles must deal with several barriers in order to be able to interact with DNA, the nuclear membrane being the most relevant of these. Size is a very relevant feature that can notably affect the localization of the nanoparticle inside the cell. Although small molecules (<9 nm in diameter) seem to be able to enter the nucleus without regulation, larger molecules (>30 nm in diameter) require association with importins to be internalized in the nucleus via an active process [78].

This characteristic becomes highly relevant, since any molecule or particle intended to enter into the nucleus must present a nuclear localization signal (NLS) to interact with the nuclear pore complex, and it must be smaller than 30 nm to cross the nuclear membrane [79]. However, some studies also point out that the nuclear localization of Au NPs can be size-dependent, showing a nuclear accumulation of small NPs (3 and 10 nm in size) even without NLS. Moreover, 25 and 50 nm particles accumulated around the nucleus [80]. Related to this, it has been reported that 1.4 nm size Au_55_ (radioactive gold isotope) clusters are able to enter the nucleus, where they interact irreversibly with the major grooves of DNA, binding DNA and nuclear proteins, inducing a cytotoxic effect [81]. Likewise, tiopronin-covered gold nanoparticles (Au-TIOP NPs) smaller than 10 nm (2 and 6 nm) were able to enter the nucleus of MCF-7 breast cancer cells, whereas larger ones (10 and 16 nm) were found only in the cytoplasm [82].

Nevertheless, in general, it is convenient for nanoparticles to incorporate the NLS sequence in order to interact with the nuclear pores. A study using human oral squamous cell carcinoma (HSC) confirms that 30 nm citrate Au NPs were not able to localize at the nucleus. However, when they were conjugated with an NLS peptide, their nuclear internalization was possible, causing cytokinesis arrest, leading to DNA damage and the failure of cell division, and resulting in apoptosis [83]. Likewise, gold nanospheres and nanocages (34 and 44 nm, respectively) conjugated with NLS and also with arginine–glycine–aspartic acid (RGD) peptides were also able to penetrate the nucleus, inducing cell cycle changes and reducing ATP production, which led to apoptosis and necrosis [84]. Other examples of internalization include the conjugation of gold nanorods smaller than 50 nm with an NLS peptide that allowed them to enter the nucleus of both HaCat and HSC 3 cells [85] and the nuclear translocation of Au NPs (20 nm) conjugated with adenoviral NLS and receptor-mediated endocytosis (RME) peptides into the nucleus of HepG2 cells when they are conjugated with both RME and NLS peptides [79].

In general, conjugation with RGD and/or NLS peptides seems to be an appropriate strategy to allow the internalization of nanomaterials into the nucleus [78,84,86]. However, it is worth noting that this phenomenon has also been observed in nanomaterials coated with RALA, a short amphipathic peptide [87,88], as well as when functionalized with 3-mercaptopropionic acid (MPA) and NH2-PEG-NH2 [89] and dimethyl-dioctadecyl-ammonium bromide (DODAB) [90].

A summary of the effects generated by internalized Au NPs is presented in Table 4.

### 3.4. SiO_2_ NPs (Silica NPs)

SiO_2_ NPs have been proposed as potential nanocarriers of DNA molecules [91], thus being critical in determining the interaction between these NPs and the DNA. The predictive model proposed by Li et al. predicts that SiO_2_ NPs do not bind to the DNA [10]; however, molecular dynamic simulations performed by Shi et al. describe two major binding mechanisms to explain DNA-SiO_2_ NP binding: (1) attractive interactions between DNA phosphates and (2) surface silanol groups and hydrophobic bonding between the DNA base and the silica hydrophobic region [92]. Additionally, a third mechanism could be considered, as cationic lipids have a high propensity to bind DNA molecules in vitro [91,93].

The interaction of SiO_2_ NPs with DNA in a biological system, as we have previously described, strongly depends on the ability of the NPs to cross the nuclear membrane. In this context, 70 nm nonporous amorphous silica particles have been reported to enter the nucleus of HaCaT cells (where they showed high ROS generation and DNA damage) as well as the nucleus of mice skin cells, cervical lymph node cells and parenchymal hepatocytes. The same phenomenon occurred in Langerhans cells and murine macrophages. ROS generation and DNA damage have also been observed after treatment, and it has been hypothesized that size-dependent effects might take place due to intracellular uptake mechanisms. It has also been theorized that 70 nm SiO_2_ NPs might interact with nuclear transporting proteins and that SiO_2_ NPs/protein complexes are transported into the nucleus [94,95,96,97]. The presence of fluorescent SiO_2_ NPs has also been previously reported with sizes of 40 and 70 nm, inside the nucleus of HEp-2 cells, the latter inducing aberrant nucleoplasmic protein aggregation [98]. Another related work studies the increased nuclear uptake of 28 nm SiO_2_ NPs (called LumiLys 650) in the nucleus of HCT-116 and RT cells after electroporation [99]. With respect to this phenomenon, electroporation can induce a 70% increase in cell nucleus size, thus potentially increasing the size of nuclear pores [100].

Regarding the potential oxidative stress caused by SiO_2_ NPs, treatment with 43 and 62 nm SiO_2_ NPs in human umbilical vein endothelial cells (HUVECs [101] and HepG2 [102], respectively) causes oxidative stress, leading to DNA damage response, provoking toxicity through the G2/M DNA damage checkpoint signaling pathway [101], and leading to mitochondrial potential decreasing and apoptosis through mitochondrial pathway activation [102]. In addition, increased ROS levels have also been observed in a dose- and size-dependent way in HEpG2 cells, where a size reduction in the NPs leads to higher ROS production, the 19 nm SiO_2_ NPs being the most toxic of all the sizes studied [103].

A summary of the effects generated by SiO_2_ NPs is given in Table 5.

### 3.5. Other Nanomaterials

#### 3.5.1. Nanosized-Metal Organic Frameworks (NMOFs)

Metal organic frameworks, commonly known as MOFs, have arisen lately as a potential class of materials for diverse applications, such as drug delivery, radio enhancement, imaging or theranostics [104]. MOFs are catalogued as micro- or meso-porous crystalline materials, which consist of metal ions, clusters or chains interconnected by organic linkers [105]. In this context, these materials have been transferred to the nanometric scale, generating nanosized-MOFs (or NMOFs), which present several interesting features such as malleable morphology, size or composition, while preserving MOF’s characteristic properties, i.e., large surface area or high porosity [106]. In particular, their higher quantity of active sites due to their large surface area makes them ideal nanocarriers for smart drug delivery [107].

All the features described above make this kind of nanomaterial a highly plausible alternative for developing sophisticated antitumoral therapies. In particular, some of these NMOFs exhibit anticancer activity by, among others, producing alterations in the genetic material. In this context, an iron-based NMOF has been developed, combined with the addition of dihydroartemisinin (DHA). The interaction of leached iron ions with DHA generated a substantial quantity of toxic ·OH which, via ferroptosis triggering, eventually led to DNA damage, further confirming therapeutic efficacy in vivo [108]. In a similar way, complete manganese based-NMOF that leads to a reduction in GSH levels and thus, an increase in cytoplasmic and mitochondrial ROS generation, which led to DNA damage, has been developed [109].

Some NMOFs have also demonstrated the capacity of causing DNA damage upon some external stimulation, i.e., in the case of radiosensitizer NMOFs, or NMOFS used for photodynamic therapy (PDT) or photothermal therapy. As an example, an NMOF based on Hf and Mn was able to successfully combine PTT and X-ray therapy and caused an increase in DNA damage upon X-ray irradiation [110]. Likewise, some NMOFS, as it was mentioned before, are extremely suitable for the delivery of certain. That is the case for a biocompatible Zr-based NMOF, which presented a high loading capacity of doxorubicin (DOX). The correct delivery of this drug helped to increase the apoptotic and cell cycle alterations in HepG2 cells [106].

Unfortunately, and despite the multiple applications already mentioned, this kind of nanomaterial is not exempt from potential drawbacks. The most important concerns are the potential triggering of undesired genotoxic and cytotoxic effects, which have been strongly correlated with nMOF composition, regarding the metal and organic building blocks. Each metal possesses a particular lethal dose, and so do the organic building blocks, which should be considered when designing NMOFs [111]. Likewise, other elements related to the preparation of NOMFs, such as organic linkers or the solvent, have demonstrated a strong influence on the potential cytotoxic effects derived from NMOFs administration, making it necessary to take these factors into account [112]. Furthermore, other crucial aspects such as the genotoxicity of these materials should be more frequently revised in order to elucidate the potential of DNA damage.

In addition, it has been recognized that these materials present problems of stabilization mainly compared to metallic nanoparticles. Although several approaches for overcoming this issue are being implemented, the implications are yet mostly unknown, and their reaction products and transformations are not fully characterized. Thus, this lack of information may have implications in the toxicity and activity of these materials in the biological medium [104]. In this context, Herrmann’s laboratory assessed the stability of several NMOFs. The behavior of TiZr-PCN NMOFs, which initially showed high stability in different buffers, was of particular interest. However, the partial segregation of Ti and Zr was detected after 2 months of cell culture, which is an issue to be concerned about, since some studies suggest DNA could be a possible intracellular target for Ti [104]. Other studies have also attributed NMOFs toxicity to the solubility and subsequent released metal ions [113]. Related to this, it is remarkable that some studies are in fact concerned about this issue. Cai et al. designed a biodegradable, copper-based NMOF, studying the degradation profile of this material in an in vivo assay with mice. The results showed that, in fact, 90% of the nanomaterial was excreted via urine and feces within 30 days [114].

Finally, it is worth noting that, as it was previously mentioned, different strategies are being studied in order to overcome the limitations of NMOFs that have been described so far. In particular, biomimetics has produced some good results in the field of nanobiomedicine, and NMOFs are no exception. Studies with the cloaking of different cell membranes, such as cancer cells or erythrocytes, have provided immunity escape and preferential accumulation to different MOFs [115]. Thus, the development of this strategy could be an important step in the consolidation of NMOFs as a solid, effective and specific therapy in the treatment of several diseases.

#### 3.5.2. Magnetic Nanometer-Size Particles

Magnetic nanoparticles (MNPs) are a class of nanomaterials composed of metals with paramagnetic, ferromagnetic, or superparamagnetic properties, such as cobalt, nickel, and iron [116]. The MNPs have demonstrated great efficacy as thermoelectric materials, imaging agents, drug delivery vehicles, and biosensors [117]. The most commonly used MNP is the superparamagnetic magnetite iron oxide (Fe_3_O_4_) because of its high biocompatibility and low toxicity; nevertheless, recently, iron oxide MNPs, with smaller sizes, have been designated as the best choice for biological and biomedical applications [118].

DNA molecules have a number of metal-ion binding sites. There are oxygen atoms of phosphate groups, hydroxyl groups of sugars, endocyclic nitrogen atoms and exocyclic keto groups of bases. Interactions of metal ions with different sites of DNA molecules are specific and depend strongly on the nature of the ions. Fe2+ cation is directly coordinated to the N(7) atom of guanine and indirectly, through water molecules, to the O(6) atom of guanine and phosphate groups, thus forming an octahedral coordination complex of iron. NPs bind to DNA molecules through the formation of Fe7O7P bonds. Additionally, the interaction of nanoparticles with the double-stranded DNA induces the cross-linking of phosphate groups, which led to DNA compaction [118].

MNPs have a high surface area to volume ratio, high binding rate with detection substances, and can perform magnetically controllable aggregation and dispersion, making preconcentration, purification and the separation of nucleic acids simple and easy; therefore, MNPs can be employed for nucleic acid extraction, target enrichment, infectious disease identification, site mutation detection, and library preparation for next-generation sequencing [119]. These applications have been widely explored; however, new applications can be developed such as the differentiation of mesenchymal cells into chondrocytes, adipocytes and osteoblasts on substrates with nanotopography generated by MNPs and DNA, allowing the differentiation of the cells and reducing the necessity of growth factors [120].

#### 3.5.3. Lipid-Based Nanoparticles (LPB NPs)

In recent decades, lipid-based nanoparticles (LPB NPs) have also attracted attention because of their properties as molecule carriers, particularly DNA and RNA. DNA therapy allows gene delivery, whereas RNA therapy offers the possibility to knock down, insert or replace a disease-associated DNA [121,122,123]. However, with regard to nucleic acids, LPB NPs can act in more ways than simply as a carrier. The size of the cationic LPB NPs varies depending on the carried DNA, and among them, DOTMA/DOPE liposomes, some of the most widely used types of cationic LPB NPs, are usually large NPs which are internalized and retained in the cytoplasm surrounding the nucleus [124]. Therefore, in the case of this nanomaterial, the generation of DNA damage might consist of an indirect process via inflammation and oxidative stress [125,126,127]. Cationic LPB NPs are able to induce DNA breaks in mice, specifically in the lung, liver and kidney, in a dose-dependent manner. These nanoparticles also induce DNA breaks and later repair in human peripheral blood cells in vitro, in this case one hour after treatment [128]. In another study, it was observed that that the injection of standard cationic non-PEGylated DOTAP/CHOL liposomes (92 nm) in Wistar rats, in a single or in consecutive intravenous doses, increased DNA damage in the lung and spleen. The administration of these liposomes did not induce detectable DNA breaks in the liver, although they induced the expression of proinflammatory cytokines, stress response gene HOMX1 and DNA repair enzyme OGG1 [127]. It has also been reported that cationic liposomes and cationic DOTAP also induce in a dose-dependent manner an increase in reactive oxygen intermediates (ROIs) and pulmonary inflammation in mice [125]. Likewise, it has been shown the cationic DOTAP liposome is an active stimulator of mouse bone marrow-derived dendritic cells (BMDCs), resulting in the activation of ERK and p38 and the induction of chemokines, cytokines and co-stimulatory molecules mediated by appropriate amounts of ROS [126].

#### 3.5.4. Nanoparticle Pollution

Diesel exhaust particles (DEPs) are generated from the incomplete combustion of diesel fuel, being able to cause oxidative DNA damage, which is associated with polymorphisms that confer deficiencies in DNA repair and/or xenobiotic metabolic pathways in white blood cells [129]. In addition to DNA damage, these NPs can modify the methylation profile, which plays an important role in the reprogramming of gene expression. To date, only two studies focusing on the analysis of DNA methylation in real human populations occupationally exposed to NP have been published. The first study assessed global methylation and oxidative DNA damage in workers with occupational exposure to metal oxide nanomaterials, and it revealed that exposure to SiO_2_ and indium tin oxide NPs significantly increased oxidative biomarkers and global DNA hypomethylation levels [130]. The second study analyzed the inhalation exposure of workers to NPs during the nanocomposite producing processes (welding, machining). Results show that short-term exposure induces DNA damage without disturbing DNA methylation, although long-term exposure leads to the adaptation of the epigenome by DNA methylation modification without detectable DNA damage. Differences in CpG methylation levels between the exposed and control subjects were detected in signaling-pathways-related genes, including cytokines, genes involved in lung functions, cancer, blood cell count and lipid metabolism, xenobiotic detoxification, cognitive functions and type II diabetes [131].

## 4. Conclusions

Nanomaterials have unique properties that make them useful for a wide range of applications in biomedicine, including diagnosis, treatment and theragnosis. The development and improvement of these materials for their use in biomedicine has increased over the past years. However, their use is accompanied with increasing concerns regarding the biosafety of these materials, thus limiting the transfer of nanomaterials to the clinics.

For this reason, more extensive studies are needed to understand the role of nanomaterials in the metabolism of other compounds as well as ensure the publication of relevant information on the biosecurity of the nanomaterials tested. A clear example of how these materials can disrupt several metabolic pathways is derived from the production of ROS. Several nanomaterials have been related with ROS production and DNA damage induction. Likewise, the potential genotoxic effect of some nanomaterials must not be overlooked.

Consequently, there is a need for the development of accepted and specific protocols to identify the entire metabolic alteration of each nanomaterial regardless of their application. This standardization would enhance our knowledge, leading to the safer use of these materials, and increasing the number of available NPs for the wide variety of biomedical applications where novel and groundbreaking therapies are urgently required.

## Figures and Tables

**Table 1:** Effect of AgNPs on DNA.

Size	Stabilizer/Coating	Cell Line/Animal Model	Effect	Ref.
6–20 nm	Sodium borohydride reduction Starch-capped	U251-MG, IMR-90	Mitochondrial disfunction and increase in ROS production. DNA damage and chromosomal aberrations. Cell cycle arrest.	[24]
5 nm	Commercial NPs (I&C Technology, Seoul, Republic of Korea) PVP-coated	HepG2, Huh7, THP-1	Reduction in nuclear factor erythroid 2-like 2 expression after treatment with 5 nm Ag NPs in both hepatoma cell lines. Increase in ROS production after treatment with 5 nm Ag NPs in hepatoma cell lines Alteration of glucose metabolism after treatment with 5 nm Ag NPs in hepatoma cell lines and THP-1 cells.	[26]
100 nm				
20 nm	Commercial NPs (Shanghai YunfuNano Technology Co., Ltd., Shanghai, China)	HT22	Cell viability reduction and membrane leakage induction in a dose-dependent manner. Increase in ROS production. Autophagy induction, upregulation of LC3 II/I, downregulation of p62. Upregulation of caspase-3 and Bax, downregulation of Bcl-2. Alteration of PI3K/AKT/mTOR signaling pathway.	[27]
43–260 nm	Commercial NPs (Sigma Aldrich, St. Louis, MO, USA)	BEAS-2B	Stimulation of DNA breakage and micronuclei formation. Induction of increased oxidative DNA damage. Increase in reactive oxygen radicals.	[28]
20 nm	Commercial NPs (Plasmachem GmbH, Berlin, Germany)	Ntera2 (NT2, human testicular embryonic carcinoma cell line)	Cytotoxic and cytostatic. Apoptosis and necrosis. Decreased proliferation. Concentration- and time-dependent manner. 200 nm Ag NP increase DNA-strand breaks.	[29]
200 nm				
10 nm	Commercial NPs (NanoComposix, San Diego, CA, USA)	Jurkat Clon E6-1, THP-1	Micronuclei and DNA damage inversely correlated with Ag NP size. Suggestion that silver ions are the main cause of NP genotoxicity.	[30]
20 nm				
50 nm				
100 nm				
5 nm	Commercial NPs (NanoComposix, San Diego, CA, USA) PVP-coated	TK6	Genotoxicity and cytotoxicity induction in a range of concentrations similar to silver nitrate (AgNO_3_). Induction of oxidative stress. Suggestion that silver ions are not the cause of NP genotoxicity.	[31]
42.5 nm	Commercial NPs (NANOGAP, Milladoiro, Spain) PVP-coated	BEAS-2B	DNA damage induction in a dose-dependent manner. No induction of micronuclei or chromosomal aberrations observed. Lack of chromosomal damage may be due to PVP-coating protection from leaching or direct interaction with Ag NPs.	[32]
5 nm	Commercial (NanoComposix, San Diego, CA, USA) PVP-coated	B6C3F1 mice—intravenous administration	Increase in oxidative damage with PVP- and silica-coated NPs of different sizes. Induction of toxicity in bone marrow caused by PVP-coated NPs but not by silica-coated NPs. No significant increase in mutant frequencies in the *Pig*-a gene or the percent of micronucleated reticulocytes. Induction of oxidative DNA damage in liver by PVP- and silicon-coated NPs.	[33]
15–100 nm				
10–80 nm	Commercial (NanoComposix, San Diego, CA, USA) Silicon-coated			
6.3–629 nm	Commercial NPs (Nanux, SL1105001, Gimhae, Republic of Korea)	Sprague-Dawley rats—intravenous administration	Highest Ag concentrations found in lung, spleen and liver. Marked increase in alanine aminotransferase (ALT), blood urea nitrogen (BUN), total bilirubin (TBil) and creatinine (Cre) after NP administration. Induction of extensive organ damages in liver, kidneys, thymus and spleen after NP administration. Increase in aberration and multiple aberrations cells, as well as polyploidy cells after NP administration.	[34]
14 nm	Hydrazine reduction PVP-coated	Female Wistar Hannover Galas rats—oral administration	Largest silver concentrations found in intestinal system, liver and kidneys. Lower concentrations of absolute silver after administration of NPs than silver acetate (AgAc). Sulfur and selenium containing silver granules found in lysosomes of macrophages of the ileum.	[35]
15 nm	Commercial (Collargol, Laboratorios Argenol S.L, Zaragoza, Spain.)	Weanling male Sprague–Dawley rats	Significant accumulation of silver found in liver and kidneys. Presence of Ag(I) complexed by high-molecular proteins in liver.	[36]

**Table 2:** Effect of CeO_2_ NPs on DNA.

Size	Main Features	Cell Line/Animal Model	Main Effects and Conclusions	Ref.
8–20 nm	Commercial NPs (Sigma Chemical Co., Ltd., St. Louis, MO, USA)	A549	ROS generation, reduction in GSH concentration. Nuclear fragmentation and chromatin condensation induction. Apoptotic bodies generation. Caspase-3 and caspase-9 increased expression. PARP-1 cleavage and p53 phosphorylation.	[46]
38 nm	Commercial NPs (Sigma Aldrich, St. Louis, MO, USA)	A375	ROS generation, reduction in GSH concentration. Chromatin condensation induction. DNA double-strand break formation. Caspase-3 activity induction.	[47]
7 nm	Commercial NPs (Rhodia Chemicals, Briton, UK)	Human spermatozoa fr0m healthy fertile donors	Significant induction of DNA damage. Genotoxicity inversely proportional to the concentration. Accumulation along the flagellum and no internalization in spermatozoa.	[48]
7 nm	Commercial NPs (Rhodia Chemicals, Briton, UK)	Primary human foreskin fibroblasts	Micronuclei formation. Induction of lipid peroxidation. Intracellular GSH/GSSG ratio decrease. Clastogenic mechanism of chromosomal damage.	[49]
20 nm	Commercial NPs (Sigma Aldrich, St. Louis, MO, USA) Intratracheal administration	BALB/C mice—intratracheal (IT) administration	ROS levels significantly increased in lung, heart, kidneys and brain. DNA damage in lung, heart, liver, kidneys, spleen and brain. Interleukin-6 (IL-6) increment in lung, heart, liver, kidneys, and spleen. Interleukin-1*β* (IL-1*β*) increment in lung, heart, kidneys, and spleen.	[50]
		Male Wistar rats (acute kidney injury model)—IT administration	Significant exacerbation of DNA damage after treatment with NPs. Increment in Nrf2 expression in cardiac myocytes and endothelial cells. Elevation of coagulation function, troponin I, lactate dehydrogenase, IL-6 and TNFα in plasma. Increment in renal injury molecule-1, IL-6, tumoral necrosis factor α (TNFα) and glutathione concentrations in kidneys. Inhalation of nanomaterial has more serious effects in individuals with renal diseases.	[39,51]
		Male Wistar rats (vascular damage model)—IT administration	Increment in DNA damage in aortic tissue and aggravation of vascular toxicity. Increment in Nrf2 expression in the nuclei of smooth muscles and endocardial cells	[52]
24 nm	Commercial NPs (Sigma Chemical Co., Ltd., St. Louis, MO, USA)	Albino Wistar rats—oral administration	Increment in DNA damage in liver and peripheral blood leukocytes. Increment in micronuclei and chromosomal aberrations in bone marrow. Increment micronuclei in peripheral blood.	[53]
9.5 nm	Commercial NPs (Sigma Aldrich, St. Louis, MO, USA)	BEAS-2B	Reduction in oxidative stress and DNA damage caused by KBrO_3_. Downregulation of the expression of *Ho1* and *Sod2* genes.	[54]
25 nm	Commercial NPs (Sigma Aldrich, St. Louis, MO, USA)	Albino Westar rat—intraperitoneal administration	Alleviation of DNA fragmentation in liver caused by D-GALN/LPS-induced hepatotoxicity. Nrf-2 translocation and HO-1 gene expression decrease. Increment in GSH, GPX1, glutathione reductase, superoxide dismutase and catalase.	[55]
70 nm	EU Joint Research Center (NM212)	Caco-2	No induction of cytotoxic or genotoxic effects of NPs.	[56]
10–30 nm	Octahedron-like hydrothermal synthesis	HepG2	Cytotoxic effect inversely proportional to surface area: apoptosis induction; mitochondrial membrane potential (MMP), ROS and GSH increase, and reduction in cell ability to scavenge hydroxyl free radicals. Protection and inhibition of DNA damage by scavenging with octahedron-like and rod-like NPs.	[57]
20–50 nm	Cube-like hydrothermal synthesis			
8 nm diameter 100–400 nm length	Rod-like Hydrothermal synthesis			
23 nm	Commercial NPs (Sigma Aldrich Chemical Company, St. Louis, MO, USA)	Swiss albino mice—oral administration	Amelioration of genotoxicity induced by lead acetate. Significant reduction in tail length, DNA% in tail, tail moment and percentage of fragmented DNA. Possible antioxidant capacity of NPs due to reversible switching between III and IV oxidation states.	[58]
9 nm (D_H_)	Thermo-hydrolysis synthesis Polymer coating (MPEG_2K_-MPh, MPEG_2K_-MPEGa_1K_-MPh and MPEG_2K_-MPEGa_2K_-MPh)	bEnd.3	Reduction in glutamate-induced intracellular production of ROS in endothelial cells by all NPs. Coated NPs are devoid of cytotoxicity. Lack of toxicity corroborated in vivo (male Swiss mice).	[59]
27.2 nm (D_H_)				
29.5 nm (D_H_)				
31.5 nm (D_H_)				
10 nm	Wet-chemical synthesis	Jurkat	Prevention of DNA damage and ROS production after UV-A irradiation. Prevention of micronuclei formation after UV-B irradiation.	[60]
3–5 nm	Wet-chemical synthesis	hBMSCs	Reduction in ROS levels and DNA damage after irradiation. Increase in autophagy and bone matrix deposition after irradiation.	[61]
<25 nm	Commercial NPs (Sigma Aldrich, St. Louis, MO, USA)	MC3T3-E1 osteoblast-like cells	Attenuation of deteriorative effects of irradiation, alleviating cell viability, differentiation and mineralization. Alleviation of intracellular ROS production and extracellular hydrogen peroxide (H_2_O_2_) concentration.	[62]
5–8 nm	Commercial NPs (Sigma Aldrich, St. Louis, MO, USA)	C57BL/6J male mice—intravenous administration	Significant reduction in tissue damage caused by irradiation. Substantial decrease in DNA damage and ROS. Sperm protection.	[63]

**Table 3:** Effect of Au NPs on DNA.

Size	Stabilizer/Coating		Cell Line/Animal Model	Effects	Ref.
10.9 nm	Poly(amidoamine) (PAMAM)		HepG2, PBMC	Interaction of both NPs with both cell lines and exhibition of geno- and cytotoxicity. Observation of higher sensitivity of HepG2 to Au NPs than PBMC.	[68]
18.2 nm	Citrate				
18.4 nm	Citrate		HepG2	No induction of significant cytotoxicity by both Au NPs. DNA damage production after treatment with citrate Au NPs but not after MUA-AuNP treatment.	[69]
	11-mercaptoundecanoic acid (MUA)				
18 nm	Citrate	Bovine Serum Albumin (BSA)	HepG2	Differences in the biochemical effects produced by Au NPs depending on their coating. No overall toxicity of citrate Au NPs. Induction of DNA damage that could not be repaired by citrate Au NPs. Unbound citrate shows high toxicity.	[70]
		Poly(sodium 4-styrene sulfonate)			
		Citrate			
		MUA			
		GSH			
		PVP			
		Polyethylene Glycol (PEG)			
14 nm	Citrate		Caco-2, HaCaT	Significant increase in micronuclei and nucleoplasmic bridge production at 5 nM concentration for both citrate NPs and both cell lines. Induction, by all tested AuNPs, of genotoxicity, indicating DNA damage. Induction of highest level of toxicity for 14 nm citrate Au NPs.	[64]
20 nm					
14 nm	PEG	COOH ligand			
		NH_2_ ligand			
		OH ligand			
14 nm 20 nm	Citrate		CHO	No statistical significance in the genotoxic effect observed for citrate Au NPs at the tested concentrations.	[72]
10 nm 30 nm	Citrate		Wistar rats—acute and chronic intraperitoneal administration	Increase in the frequency of DNA damage and the damage index in blood and liver. DNA damage caused by of oxidative stress regardless of the type of administration and Au NP size.	[74]
				Au NPs induce DNA damage differently depending on the type of administration and the size of the Au NPs. There are higher levels of damage frequency and damage in the DNA by 30 nm Au NPs compared to 10 nm Au NPs.	[75]

**Table 4:** Effect of internalized Au NPs on DNA.

Size	Stabilizer/Coating		Cell Line	Effect	Ref.
15 nm	Citrate	RGD peptide	HeLa	Five-fold increase in NPs uptake and effective nuclear localization for peptide-capped Au NPs. Stabilization of conjugated Au NPs complex thanks to pentapeptide. Reduction in NPs exocytosis for peptide-capped NPs compared to citrate-capped ones.	[78]
		NLS peptide			
		Pentapeptide			
35 nm	Citrate	mPEG-SH-5000 RGD peptide NLS peptide	HSC-3	Co-localization of RGD/NLS-conjugated nanomaterials with the nucleus by confocal imaging. Induction of cell cycle changes and reduced ATP production when cells are treated with RGD/NLS-conjugated nanomaterials. Higher induction of apoptosis and necrosis by RGD/NLS-conjugated hollow gold nanocages than by peptide-conjugated solid gold nanospheres.	[84]
45 nm	PVP				
30 nm	Citrate	mPEG-SH-500 RGD peptide	HSC-3	Dark-field light scattering confirmation that NLS-Au NPs were localized at the cell nucleus, while RGD-Au NPs were distributed throughout the cytoplasm.	[86]
		mPEG-SH-500 NLS peptide			
15 nm	Citrate	RALA peptide	PC-3	Decoration with RALA peptide imparts nuclear targeting capabilities. Precise 3D location of Au NPs within the cell, showing evidence of the nuclear uptake of monodispersed Au NPs. 79% of RALA-Au NPs internalized by the nucleus are predicted to be monodispersed nanoparticles.	[87]
15 nm	Citrate	RALA peptide	PC-3, DU145, PNT2-C2	RALA stabilization of sub-110 nm complexes of several Au NPs. Validation of nuclear accumulation of RALA-Au NPs. Meaningful radiosensitization of cells using low microgram RALA-AuNP concentrations.	[88]
4 nm	Citrate	PEG (MW 2000) MPA	HeLa	No induction of obvious cytotoxicity. Time-dependent cellular uptake of AuNP@MPA-PEG. Improvement of stability and biocompatibility due to surface modification. Potential nuclear-targeted drug delivery carrier.	[89]

**Table 5:** Effect of SiO_2_ NPs on DNA.

Size	Stabilizer/Coating	Cell Line/Animal Model	Effect	Ref.
70 nm	Commercial NPs (Micromod Partikeltechnologie GmbH, Rostock, Germany)	HaCat Raw246.7 Langerhans cell-like (XS52) BALB/c mice—Application in the inner side of both ears	Internalization of 70 nm SiO_2_ NPs in nucleus of HaCat cells, mice skin cells, cervical lymph node cells and parenchymal hepatocytes. Same observation for Langerhans cells and murine macrophages. Higher ROS generation after 70 nm SiO_2_ NPs in HaCat cells compared to the other sizes. DNA damage.	[94,95,96,97]
300 nm				
1000 nm				
50 nm	Commercial NPs (Kisker (Steinfurt, Germany) and Postnova (Landsberg/Lech, Germany).)	HEp-2, A549, RLE-T6N, N2a	Nuclear localization observed in HaCat cells treated with 40 and 70 nm SIO_2_ NPs. Induction of aberrant nucleoplasmic protein aggregation in HEp-2 cells after treatment with 70 nm SiO_2_ NPs.	[98]
70 nm				
200 nm				
500 nm				
1000 nm				
5000 nm				
28 nm (LumiLys 650)	Commercial NPs (Chromalys, Toulouse, France) Functionalization with gadolinium—diethylene–triamine–pentaacetic acid)	HCT-116, RL	Nucleus internalization of LumyLys 650 NPs in both cell lines after electropermeabilization. Tumor cell tracking for 30 days, labeling cells with SiO_2_ NPs by electropermeabilization.	[99]
30 nm (LumiLys 780)				
62 nm	Stöber method (tetraethyl orthosilicate (TEOS) + ethanol/ammonia/water)	HUVECs	Induction of ROS generation and DNA damage response, causing endothelial cells toxic effect through Chk-1 dependent G2/M DNA damage checkpoint signaling pathway.	[101]
43 nm	Stöber method (TEOS + ethanol/ammonia/water)	HepG2	SiO_2_ NP induction of oxidative stress through ROS production, causing mitochondrial membrane potential decrease and apoptosis through mitochondrial pathway.	[102]
19 nm	Provided by School of Chemistry, Jilin University, Changchun, China	HepG2	Toxicity produced by SiO_2_ NPs was in a dose- and size-dependent manner. The smaller the size, the higher the ROS production. All four SiO_2_ NPs led to DNA damage, cell cycle arrest and apoptosis.	[103]
43 nm				
68 nm				
498 nm				

## Data Availability

Not applicable.

## References

[1] Feynman R.P. (1960). There’s Plenty of Room at the Bottom—An invitation to Enter a New Field of Physics. Eng. Sci..

[2] Cheng Z., Li M., Dey R., Chen Y. (2021). Nanomaterials for cancer therapy: Current progress and perspectives. J. Hematol. Oncol..

[3] Shafiq M., Anjum S., Hano C., Anjum I., Abbasi B.H. (2020). An Overview of the Applications of Nanomaterials and Nanodevices in the Food Industry. Foods.

[4] Mazari S.A., Ali E., Abro R., Khan F.S.A., Ahmed I., Ahmed M., Nizamuddin S., Siddiqui T.H., Hossain N., Mubarak N.M. (2021). Nanomaterials: Applications, waste-handling, environmental toxicities, and future challenges—A review. J. Environ. Chem. Eng..

[5] Pelaz B., Alexiou C., Alvarez-Puebla R.A., Alves F., Andrews A.M., Ashraf S., Balogh L.P., Ballerini L., Bestetti A., Brendel C. (2017). Diverse Applications of Nanomedicine. ACS Nano.

[6] Martin-Pardillos A., Martin-Duque P. (2023). Cellular Alterations in Carbohydrate and Lipid Metabolism Due to Interactions with Nanomaterials. J. Funct. Biomater..

[7] Mohammadinejad R., Moosavi M.A., Tavakol S., Vardar D.O., Hosseini A., Rahmati M., Dini L., Hussain S., Mandegary A., Klionsky D.J. (2019). Necrotic, apoptotic and autophagic cell fates triggered by nanoparticles. Autophagy.

[8] Delgado D., del Pozo-Rodriguez A., Solinis M.A., Rodriguez-Gascon A. (2011). Understanding the mechanism of protamine in solid lipid nanoparticle-based lipofection: The importance of the entry pathway. Eur. J. Pharm. Biopharm..

[9] Cooper G.M. (2000). The Cell: A Molecular Approach.

[10] Li K., Zhao X., Hammer B.K., Du S., Chen Y. (2013). Nanoparticles Inhibit DNA Replication by Binding to DNA: Modeling and Experimental Validation. ACS Nano.

[11] Khan S.T., Malik A., Wahab R., Abd-Elkader O.H., Ahamed M., Ahmad J., Musarrat J., Siddiqui M.A., Al-Khedhairy A.A. (2017). Synthesis and characterization of some abundant nanoparticles, their antimicrobial and enzyme inhibition activity. Acta Microbiol. Immunol. Hung..

[12] Gao C.H., Mortimer M., Zhang M., Holden P.A., Cai P., Wu S., Xin Y., Wu Y., Huang Q. (2019). Impact of metal oxide nanoparticles on in vitro DNA amplification. PeerJ.

[13] Chen T., Yan J., Li Y. (2014). Genotoxicity of titanium dioxide nanoparticles. J. Food Drug Anal..

[14] Zielinska A., Carreiro F., Oliveira A.M., Neves A., Pires B., Venkatesh D.N., Durazzo A., Lucarini M., Eder P., Silva A.M. (2020). Polymeric Nanoparticles: Production, Characterization, Toxicology and Ecotoxicology. Molecules.

[15] Najahi-Missaoui W., Arnold R.D., Cummings B.S. (2020). Safe Nanoparticles: Are We There Yet?. Int. J. Mol. Sci..

[16] Yang W., Wang L., Mettenbrink E.M., DeAngelis P.L., Wilhelm S. (2021). Nanoparticle Toxicology. Annu. Rev. Pharmacol. Toxicol..

[17] Yetisgin A.A., Cetinel S., Zuvin M., Kosar A., Kutlu O. (2020). Therapeutic Nanoparticles and Their Targeted Delivery Applications. Molecules.

[18] Pan Y., Neuss S., Leifert A., Fischler M., Wen F., Simon U., Schmid G., Brandau W., Jahnen-Dechent W. (2007). Size-dependent cytotoxicity of gold nanoparticles. Small.

[19] Recordati C., De Maglie M., Bianchessi S., Argentiere S., Cella C., Mattiello S., Cubadda F., Aureli F., D’Amato M., Raggi A. (2015). Tissue distribution and acute toxicity of silver after single intravenous administration in mice: Nano-specific and size-dependent effects. Part. Fibre Toxicol..

[20] Ibrahim K.E., Al-Mutary M.G., Bakhiet A.O., Khan H.A. (2018). Histopathology of the Liver, Kidney, and Spleen of Mice Exposed to Gold Nanoparticles. Molecules.

[21] Tripathy N., Hong T.K., Ha K.T., Jeong H.S., Hahn Y.B. (2014). Effect of ZnO nanoparticles aggregation on the toxicity in RAW 264.7 murine macrophage. J. Hazard. Mater..

[22] Albanese A., Chan W.C.W. (2011). Effect of Gold Nanoparticle Aggregation on Cell Uptake and Toxicity. ACS Nano.

[23] Damasco J.A., Ravi S., Perez J.D., Hagaman D.E., Melancon M.P. (2020). Understanding Nanoparticle Toxicity to Direct a Safe-by-Design Approach in Cancer Nanomedicine. Nanomaterials.

[24] AshaRani P.V., Mun G.L.K., Hande M.P., Valiyaveettil S. (2009). Cytotoxicity and Genotoxicity of Silver Nanoparticles in Human Cells. ACS Nano.

[25] Cooke M.S., Evans M.D., Dizdaroglu M., Lunec J. (2003). Oxidative DNA damage: Mechanisms, mutation, and disease. FASEB J..

[26] Lee M.J., Lee S.J., Yun S.J., Jang J.Y., Kang H., Kim K., Choi I.H., Park S. (2016). Silver nanoparticles affect glucose metabolism in hepatoma cells through production of reactive oxygen species. Int. J. Nanomed..

[27] Chang X., Wang X., Li J., Shang M., Niu S., Zhang W., Li Y., Sun Z., Gan J., Li W. (2020). Silver nanoparticles induced cytotoxicity in HT22 cells through autophagy and apoptosis via PI3K/AKT/mTOR signaling pathway. Ecotoxicol. Environ. Saf..

[28] Kim H.R., Kim M.J., Lee S.Y., Oh S.M., Chung K.H. (2011). Genotoxic effects of silver nanoparticles stimulated by oxidative stress in human normal bronchial epithelial (BEAS-2B) cells. Mutat. Res. Toxicol. Environ. Mutagen..

[29] Asare N., Instanes C., Sandberg W.J., Refsnes M., Schwarze P., Kruszewski M., Brunborg G. (2012). Cytotoxic and genotoxic effects of silver nanoparticles in testicular cells. Toxicology.

[30] Butler K.S., Peeler D.J., Casey B.J., Dair B.J., Elespuru R.K. (2015). Silver nanoparticles: Correlating nanoparticle size and cellular uptake with genotoxicity. Mutagenesis.

[31] Li Y., Qin T., Ingle T., Yan J., He W., Yin J.J., Chen T. (2016). Differential genotoxicity mechanisms of silver nanoparticles and silver ions. Arch. Toxicol..

[32] Nymark P., Catalan J., Suhonen S., Jarventaus H., Birkedal R., Clausen P.A., Jensen K.A., Vippola M., Savolainen K., Norppa H. (2013). Genotoxicity of polyvinylpyrrolidone-coated silver nanoparticles in BEAS 2B cells. Toxicology.

[33] Li Y., Bhalli J.A., Ding W., Yan J., Pearce M.G., Sadiq R., Cunningham C.K., Jones M.Y., Monroe W.A., Howard P.C. (2013). Cytotoxicity and genotoxicity assessment of silver nanoparticles in mouse. Nanotoxicology.

[34] Wen H., Dan M., Yang Y., Lyu J., Shao A., Cheng X., Chen L., Xu L. (2017). Acute toxicity and genotoxicity of silver nanoparticle in rats. PLoS ONE.

[35] Loeschner K., Hadrup N., Qvortrup K., Larsen A., Gao X., Vogel U., Mortensen A., Lam H.R., Larsen E.H. (2011). Distribution of silver in rats following 28 days of repeated oral exposure to silver nanoparticles or silver acetate. Part. Fibre Toxicol..

[36] Jimenez-Lamana J., Laborda F., Bolea E., Abad-Alvaro I., Castillo J.R., Bianga J., He M., Bierla K., Mounicou S., Ouerdane L. (2014). An insight into silver nanoparticles bioavailability in rats. Metallomics.

[37] Rodriguez-Garraus A., Azqueta A., Vettorazzi A., Lopez de Cerain A. (2020). Genotoxicity of Silver Nanoparticles. Nanomaterials.

[38] Liman R., Acikbas Y., Cigerci I.H. (2019). Cytotoxicity and genotoxicity of cerium oxide micro and nanoparticles by Allium and Comet tests. Ecotoxicol. Environ. Saf..

[39] Nemmar A., Al-Salam S., Al Ansari Z., Alkharas Z.A., Al Ahbabi R.M., Beegam S., Yuvaraju P., Yasin J., Ali B.H. (2019). Impact of Pulmonary Exposure to Cerium Oxide Nanoparticles on Experimental Acute Kidney Injury. Cell. Physiol. Biochem..

[40] Kuchma M.H., Komanski C.B., Colon J., Teblum A., Masunov A.E., Alvarado B., Babu S., Seal S., Summy J., Baker C.H. (2010). Phosphate ester hydrolysis of biologically relevant molecules by cerium oxide nanoparticles. Nanomedicine.

[41] Liu B., Liu J. (2015). Comprehensive Screen of Metal Oxide Nanoparticles for DNA Adsorption, Fluorescence Quenching, and Anion Discrimination. ACS Appl. Mater. Interfaces.

[42] Chen L., Liu B., Xu Z., Liu J. (2018). NiO Nanoparticles for Exceptionally Stable DNA Adsorption and Its Extraction from Biological Fluids. Langmuir.

[43] Link N., Brunner T.J., Dreesen I.A., Stark W.J., Fussenegger M. (2007). Inorganic nanoparticles for transfection of mammalian cells and removal of viruses from aqueous solutions. Biotechnol. Bioeng..

[44] Ferraro D., Anselmi-Tamburini U., Tredici I.G., Ricci V., Sommi P. (2016). Overestimation of nanoparticles-induced DNA damage determined by the comet assay. Nanotoxicology.

[45] Kain J., Karlsson H.L., Moller L. (2012). DNA damage induced by micro- and nanoparticles—Interaction with FPG influences the detection of DNA oxidation in the comet assay. Mutagenesis.

[46] Mittal S., Pandey A.K. (2014). Cerium oxide nanoparticles induced toxicity in human lung cells: Role of ROS mediated DNA damage and apoptosis. BioMed Res. Int..

[47] Ali D., Alarifi S., Alkahtani S., AlKahtane A.A., Almalik A. (2014). Cerium Oxide Nanoparticles Induce Oxidative Stress and Genotoxicity in Human Skin Melanoma Cells. Biochem. Biophys..

[48] Preaubert L., Tassistro V., Auffan M., Sari-Minodier I., Rose J., Courbiere B., Perrin J. (2018). Very low concentration of cerium dioxide nanoparticles induce DNA damage, but no loss of vitality, in human spermatozoa. Toxicol. In Vitro.

[49] Benameur L., Auffan M., Cassien M., Liu W., Culcasi M., Rahmouni H., Stocker P., Tassistro V., Bottero J.Y., Rose J. (2014). DNA damage and oxidative stress induced by CeO2 nanoparticles in human dermal fibroblasts: Evidence of a clastogenic effect as a mechanism of genotoxicity. Nanotoxicology.

[50] Nemmar A., Yuvaraju P., Beegam S., Fahim M.A., Ali B.H. (2017). Cerium Oxide Nanoparticles in Lung Acutely Induce Oxidative Stress, Inflammation, and DNA Damage in Various Organs of Mice. Oxidative Med. Cell. Longev..

[51] Nemmar A., Al-Salam S., Nuaman S.A., Kazim M., Mohamed F., Beegam S., Yuvaraju P., Yasin J., Ali B.H. (2021). Exacerbation of Coagulation and Cardiac Injury in Rats with Cisplatin-Induced Nephrotoxicity Following Intratracheal Instillation of Cerium Oxide Nanoparticles. Cell. Physiol. Biochem..

[52] Nemmar A., Al-Salam S., Beegam S., Yuvaraju P., Ali B.H. (2019). Aortic Oxidative Stress, Inflammation and DNA Damage Following Pulmonary Exposure to Cerium Oxide Nanoparticles in a Rat Model of Vascular Injury. Biomolecules.

[53] Kumari M., Kumari S.I., Grover P. (2014). Genotoxicity analysis of cerium oxide micro and nanoparticles in Wistar rats after 28 days of repeated oral administration. Mutagenesis.

[54] Rubio L., Annangi B., Vila L., Hernandez A., Marcos R. (2015). Antioxidant and anti-genotoxic properties of cerium oxide nanoparticles in a pulmonary-like cell system. Arch. Toxicol..

[55] Hashem R.M., Rashd L.A., Hashem K.S., Soliman H.M. (2015). Cerium oxide nanoparticles alleviate oxidative stress and decreases Nrf-2/HO-1 in D-GALN/LPS induced hepatotoxicity. Biomed. Pharmacother..

[56] Vila L., Garcia-Rodriguez A., Cortes C., Velazquez A., Xamena N., Sampayo-Reyes A., Marcos R., Hernandez A. (2018). Effects of cerium oxide nanoparticles on differentiated/undifferentiated human intestinal Caco-2 cells. Chem. Biol. Interact..

[57] Wang L., Ai W., Zhai Y., Li H., Zhou K., Chen H. (2015). Effects of Nano-CeO_2_ with Different Nanocrystal Morphologies on Cytotoxicity in HepG2 Cells. Int. J. Environ. Res. Public Health.

[58] Mohamed H.R.H. (2021). Acute Oral Administration of Cerium Oxide Nanoparticles Suppresses Lead Acetate-Induced Genotoxicity, Inflammation, and ROS Generation in Mice Renal and Cardiac Tissues. Biol. Trace Element Res..

[59] Goujon G., Baldim V., Roques C., Bia N., Seguin J., Palmier B., Graillot A., Loubat C., Mignet N., Margaill I. (2021). Antioxidant Activity and Toxicity Study of Cerium Oxide Nanoparticles Stabilized with Innovative Functional Copolymers. Adv. Healthc. Mater..

[60] Caputo F., De Nicola M., Sienkiewicz A., Giovanetti A., Bejarano I., Licoccia S., Traversa E., Ghibelli L. (2015). Cerium oxide nanoparticles, combining antioxidant and UV shielding properties, prevent UV-induced cell damage and mutagenesis. Nanoscale.

[61] Wei F., Neal C.J., Sakthivel T.S., Seal S., Kean T., Razavi M., Coathup M. (2021). Cerium oxide nanoparticles protect against irradiation-induced cellular damage while augmenting osteogenesis. Mater. Sci. Eng. C.

[62] Wang C., Blough E., Dai X., Olajide O., Driscoll H., Leidy J.W., July M., Triest W.E., Wu M. (2016). Protective Effects of Cerium Oxide Nanoparticles on MC3T3-E1 Osteoblastic Cells Exposed to X-Ray Irradiation. Cell. Physiol. Biochem..

[63] Das S., Neal C.J., Ortiz J., Seal S. (2018). Engineered nanoceria cytoprotection in vivo: Mitigation of reactive oxygen species and double-stranded DNA breakage due to radiation exposure. Nanoscale.

[64] Magogotya M., Vetten M., Roux-van der Merwe M.P., Badenhorst J., Gulumian M. (2022). In vitro toxicity and internalization of gold nanoparticles (AuNPs) in human epithelial colorectal adenocarcinoma (Caco-2) cells and the human skin keratinocyte (HaCaT) cells. Mutat. Res. Toxicol. Environ. Mutagen.

[65] Zhang X., Servos M.R., Liu J. (2012). Surface science of DNA adsorption onto citrate-capped gold nanoparticles. Langmuir.

[66] Liu Z., Hettihewa M., Shu Y., Zhou C., Wan Q., Liu L. (2018). The mechanism of the adsorption of dsDNA on citrate-stabilized gold nanoparticles and a colorimetric and visual method for detecting the V600E point mutation of the BRAF gene. Microchim. Acta.

[67] Nelson E.M., Rothberg L.J. (2011). Kinetics and mechanism of single-stranded DNA adsorption onto citrate-stabilized gold nanoparticles in colloidal solution. Langmuir.

[68] Paino I.M., Marangoni V.S., de Oliveira Rde C., Antunes L.M., Zucolotto V. (2012). Cyto and genotoxicity of gold nanoparticles in human hepatocellular carcinoma and peripheral blood mononuclear cells. Toxicol. Lett..

[69] Fraga S., Faria H., Soares M.E., Duarte J.A., Soares L., Pereira E., Costa-Pereira C., Teixeira J.P., de Lourdes Bastos M., Carmo H. (2013). Influence of the surface coating on the cytotoxicity, genotoxicity and uptake of gold nanoparticles in human HepG2 cells. J. Appl. Toxicol..

[70] Mulder D., Taute C.J.F., van Wyk M., Pretorius P.J. (2022). A Comparison of the Genotoxic Effects of Gold Nanoparticles Functionalized with Seven Different Ligands in Cultured Human Hepatocellular Carcinoma Cells. Nanomaterials.

[71] Lindeque J.Z., Matthyser A., Mason S., Louw R., Taute C.J.F. (2018). Metabolomics reveals the depletion of intracellular metabolites in HepG2 cells after treatment with gold nanoparticles. Nanotoxicology.

[72] George J.M., Magogotya M., Vetten M.A., Buys A.V., Gulumian M. (2017). From the Cover: An Investigation of the Genotoxicity and Interference of Gold Nanoparticles in Commonly Used In Vitro Mutagenicity and Genotoxicity Assays. Toxicol. Sci..

[73] Geissler D., Wegmann M., Jochum T., Somma V., Sowa M., Scholz J., Frohlich E., Hoffmann K., Niehaus J., Roggenbuck D. (2019). An automatable platform for genotoxicity testing of nanomaterials based on the fluorometric gamma-H2AX assay reveals no genotoxicity of properly surface-shielded cadmium-based quantum dots. Nanoscale.

[74] Cardoso E., Londero E., Ferreira G.K., Rezin G.T., Zanoni E.T., de Souza Notoya F., Leffa D.D., Damiani A.P., Daumann F., Rohr P. (2014). Gold nanoparticles induce DNA damage in the blood and liver of rats. J. Nanopart. Res..

[75] Cardoso E., Rezin G.T., Zanoni E.T., de Souza Notoya F., Leffa D.D., Damiani A.P., Daumann F., Rodriguez J.C., Benavides R., da Silva L. (2014). Acute and chronic administration of gold nanoparticles cause DNA damage in the cerebral cortex of adult rats. Mutat. Res. Mol. Mech. Mutagen..

[76] Lu Y., Zhang X., ZHang H., Lamn J., Huang G., Varin E., Lincet H., Poulain L., Icard P. (2011). Citrate Induces Apoptotic Cell Death: A Promising Way to Treat Gastric Carcinoma?. Anticancer Res..

[77] Uboldi C., Bonacchi D., Lorenzi G., Hermanns M.I., Pohl C., Baldi G., Unger R.E., Kirkpatrick C.J. (2009). Gold nanoparticles induce cytotoxicity in the alveolar type-II cell lines A549 and NCIH441. Part. Fibre Toxicol..

[78] Yang C., Uertz J., Yohan D., Chithrani B.D. (2014). Peptide modified gold nanoparticles for improved cellular uptake, nuclear transport, and intracellular retention. Nanoscale.

[79] Tkachenko A.G., Xie H., Coleman D., Glomm W., Ryan J., Anderson M.F., Franzen S., Feldheim D.L. (2003). Multifunctional Gold Nanoparticle-Peptide Complexes for Nuclear Targeting. J. Am. Chem. Soc..

[80] Boyoglu C., He Q., Willing G., Boyoglu-Barnum S., Dennis V.A., Pillai S., Singh S.R. (2013). Microscopic Studies of Various Sizes of Gold Nanoparticles and Their Cellular Localizations. ISRN Nanotechnol..

[81] Tsoli M., Kuhn H., Brandau W., Esche H., Schmid G. (2005). Cellular uptake and toxicity of Au55 clusters. Small.

[82] Huo S., Shubin J., Ma X., Xue X., Yang K., Kumar A., Wang P.C., Zhang J., Hu Z., Liang X.J. (2014). Ultrasmall Gold Nanoparticles as Carriers for Nucleus-Based Gene Therapy Due to Size-Dependent Nuclear Entry. ACS Nano.

[83] Kang B., Mackey M.A., El-Sayed M.A. (2010). Nuclear Targeting of Gold Nanoparticles in Cancer Cells Induces DNA Damage, Causing Cytokinesis Arrest and Apoptosis. J. Am. Chem. Soc..

[84] Mackey M.A., Saira F., Mahmoud M.A., El-Sayed M.A. (2013). Inducing cancer cell death by targeting its nucleus: Solid gold nanospheres versus hollow gold nanocages. Bioconjug. Chem..

[85] Oyelere A.K., Chen P.C., Huang X., El-Sayed I.H., El-Sayed M.A. (2007). Peptide-Conjugated Gold Nanorods for Nuclear Targeting. Bioconjug. Chem..

[86] Huang X., Kang B., Qian W., Mackey M.A., Chen P.C., Oyelere A.K., El-Sayed I.H., El-Sayed M.A. (2010). Comparative study of photothermolysis of cancer cells with nuclear-targeted or cytoplasm-targeted gold nanospheres: Continuous wave or pulsed lasers. J. Biomed. Opt..

[87] McCulloch A., Bennie L., Coulter J.A., McCarthy H.O., Dromey B., Grimes D.R., Quinn P., Villagomez-Bernabe B., Currell F. (2019). Nuclear Uptake of Gold Nanoparticles Deduced Using Dual-Angle X-Ray Fluorescence Mapping. Part. Part. Syst. Charact..

[88] Bennie L.A., Feng J., Emmerson C., Hyland W.B., Matchett K.B., McCarthy H.O., Coulter J.A. (2021). Formulating RALA/Au nanocomplexes to enhance nanoparticle internalisation efficiency, sensitising prostate tumour models to radiation treatment. J. Nanobiotechnol..

[89] Gu Y.J., Cheng J., Lin C.C., Lam Y.W., Cheng S.H., Wong W.T. (2009). Nuclear penetration of surface functionalized gold nanoparticles. Toxicol. Appl. Pharmacol..

[90] Li P., Li D., Zhang L., Li G., Wang E. (2008). Cationic lipid bilayer coated gold nanoparticles-mediated transfection of mammalian cells. Biomaterials.

[91] Castro-Smirnov F.A., Pietrement O., Aranda P., Bertrand J.R., Ayache J., Le Cam E., Ruiz-Hitzky E., Lopez B.S. (2016). Physical interactions between DNA and sepiolite nanofibers, and potential application for DNA transfer into mammalian cells. Sci. Rep..

[92] Shi B., Shin Y.K., Hassanali A.A., Singer S.J. (2015). DNA Binding to the Silica Surface. J. Phys. Chem. B.

[93] Reinhardt N., Adumeau L., Lambert O., Ravaine S., Mornet S. (2015). Quaternary ammonium groups exposed at the surface of silica nanoparticles suitable for DNA complexation in the presence of cationic lipids. J. Phys. Chem. B.

[94] Nabeshi H., Yoshikawa T., Matsuyama K., Nakazato Y., Matsuo K., Arimori A., Isobe M., Tochigi S., Kondoh S., Hirai T. (2011). Systemic distribution, nuclear entry and cytotoxicity of amorphous nanosilica following topical application. Biomaterials.

[95] Nabeshi H., Yoshikawa T., Arimori A., Yoshida T., Tochigi S., Hirai T., Akase T., Nagano K., Abe Y., Kamada H. (2011). Effect of surface properties of silica nanoparticles on their cytotoxicity and cellular distribution in murine macrophages. Nanoscale Res. Lett..

[96] Nabeshi H., Yoshikawa T., Matsuyama K., Nakazato Y., Arimori A., Isobe M., Tochigi S., Kondoh S., Hirai T., Akase T. (2010). Size-dependent cytotoxic effects of amorphous silica nanoparticles on Langerhans cells. Pharmazie.

[97] Nabeshi H., Yoshikawa T., Matsuyama K., Nakazato Y., Tochigi S., Kondoh S., Hirai T., Akase T., Nagano K., Abe Y. (2011). Amorphous nanosilica induce endocytosis-dependent ROS generation and DNA damage in human keratinocytes. Part. Fibre Toxicol..

[98] Chen M., Vonmikecz A. (2005). Formation of nucleoplasmic protein aggregates impairs nuclear function in response to SiO nanoparticles. Exp. Cell Res..

[99] Phonesouk E., Lechevallier S., Ferrand A., Rols M.P., Bezombes C., Verelst M., Golzio M. (2019). Increasing Uptake of Silica Nanoparticles with Electroporation: From Cellular Characterization to Potential Applications. Materials.

[100] Bellard E., Tessié J. (2009). Double Pulse Approach of Electropulsation: A Fluorescence Analysis of the Nucleus Perturbation at the Single Cell Level. Transactions on Dielectrics and Electrical Insulation. IEEE Trans. Dielectr. Electr. Insul..

[101] Duan J., Yu Y., Li Y., Yu Y., Li Y., Zhou X., Huang P., Sun Z. (2013). Toxic effect of silica nanoparticles on endothelial cells through DNA damage response via Chk1-dependent G2/M checkpoint. PLoS ONE.

[102] Sun L., Li Y., Liu X., Jin M., Zhang L., Du Z., Guo C., Huang P., Sun Z. (2011). Cytotoxicity and mitochondrial damage caused by silica nanoparticles. Toxicol. In Vitro.

[103] Li Y., Sun L., Jin M., Du Z., Liu X., Guo C., Li Y., Huang P., Sun Z. (2011). Size-dependent cytotoxicity of amorphous silica nanoparticles in human hepatoma HepG2 cells. Toxicol. In Vitro.

[104] Neuer A.L., Geck D., Gogos A., Kissling V.M., Balfourier A., Herrmann I.K. (2023). Nanoanalytical Insights into the Stability, Intracellular Fate, and Biotransformation of Metal-Organic Frameworks. ACS Appl. Mater. Interfaces.

[105] Zhao H., Sene S., Mielcarek A.M., Miraux S., Menguy N., Ihiawakrim D., Ersen O., Pechoux C., Guillou N., Scola J. (2023). Hierarchical superparamagnetic metal-organic framework nanovectors as anti-inflammatory nanomedicines. J. Mater. Chem. B.

[106] Fytory M., Mansour A., El Rouby W.M.A., Farghali A.A., Zhang X., Bier F., Abdel-Hafiez M., El-Sherbiny I.M. (2023). Core-Shell Nanostructured Drug Delivery Platform Based on Biocompatible Metal-Organic Framework-Ligated Polyethyleneimine for Targeted Hepatocellular Carcinoma Therapy. ACS Omega.

[107] Mosavi S.H., Zare-Dorabei R. (2022). Synthesis of NMOF-5 Using Microwave and Coating with Chitosan: A Smart Biocompatible pH-Responsive Nanocarrier for 6-Mercaptopurine Release on MCF-7 Cell Lines. ACS Biomater. Sci. Eng..

[108] Yang X.X., Xu X., Wang M.F., Xu H.Z., Peng X.C., Han N., Yu T.T., Li L.G., Li Q.R., Chen X. (2022). A nanoreactor boosts chemodynamic therapy and ferroptosis for synergistic cancer therapy using molecular amplifier dihydroartemisinin. J. Nanobiotechnol..

[109] Karim S., Halder S., Mukherjee S., Debnath U., Misra A.K., Jana K., Das D. (2023). Glutathione Depleting a Chemoselective Novel Pro-oxidant Nano Metal-Organic Framework Induced G2/M Arrest and ROS-Mediated Apoptotic Cell Death in a Human Triple-Negative Breast Cancer Cell Line. ACS Appl. Mater. Interfaces.

[110] Bao J., Zu X., Wang X., Li J., Fan D., Shi Y., Xia Q., Cheng J. (2020). Multifunctional Hf/Mn-TCPP Metal-Organic Framework Nanoparticles for Triple-Modality Imaging-Guided PTT/RT Synergistic Cancer Therapy. Int. J. Nanomed..

[111] Zhong X.F., Sun X. (2020). Nanomedicines based on nanoscale metal-organic frameworks for cancer immunotherapy. Acta Pharmacol. Sin..

[112] Shait Mohammed M.R., Ahmad V., Ahmad A., Tabrez S., Choudhry H., Zamzami M.A., Bakhrebah M.A., Ahmad A., Wasi S., Mukhtar H. (2019). Prospective of nanoscale metal organic frameworks [NMOFs] for cancer therapy. Semin. Cancer Biol..

[113] Ruyra A., Yazdi A., Espin J., Carne-Sanchez A., Roher N., Lorenzo J., Imaz I., Maspoch D. (2014). Synthesis, culture medium stability, and in vitro and in vivo zebrafish embryo toxicity of metal-organic framework nanoparticles. Chemistry.

[114] Cai X., Xie Z., Ding B., Shao S., Liang S., Pang M., Lin J. (2019). Monodispersed Copper(I)-Based Nano Metal-Organic Framework as a Biodegradable Drug Carrier with Enhanced Photodynamic Therapy Efficacy. Adv. Sci..

[115] Wen T., Quan G., Niu B., Zhou Y., Zhao Y., Lu C., Pan X., Wu C. (2021). Versatile Nanoscale Metal-Organic Frameworks (nMOFs): An Emerging 3D Nanoplatform for Drug Delivery and Therapeutic Applications. Small.

[116] Yang Y., Chawla A., Zhang J., Esa A., Jang H.L., Khademhosseini A. (2019). Applications of Nanotechnology for Regenerative Medicine; Healing Tissues at the Nanoscale. Princ. Regen. Med..

[117] Ali A., Shah T., Ullah R., Zhou P., Guo M., Ovais M., Tan Z., Rui Y. (2021). Review on Recent Progress in Magnetic Nanoparticles: Synthesis, Characterization, and Diverse Applications. Front. Chem..

[118] Pershina A.G., Sazonov A.E., Filimonov V.D. (2014). Magnetic nanoparticles–DNA interactions: Design and applications of nanobiohybrid systems. Russ. Chem. Rev..

[119] Tang C., He Z., Liu H., Xu Y., Huang H., Yang G., Xiao Z., Li S., Liu H., Deng Y. (2020). Application of magnetic nanoparticles in nucleic acid detection. J. Nanobiotechnol..

[120] Ishmukhametov I., Batasheva S., Rozhina E., Akhatova F., Mingaleeva R., Rozhin A., Fakhrullin R. (2022). DNA/Magnetic Nanoparticles Composite to Attenuate Glass Surface Nanotopography for Enhanced Mesenchymal Stem Cell Differentiation. Polymers.

[121] Dass C.R., Walker T.L., DeCruz E.E., Burton M.A. (1997). Cationic Liposomes and Gene Therapy for Solid Tumors. Drug Deliv..

[122] Ewert K.K., Zidovska A., Ahmad A., Bouxsein N.F., Evans H.M., McAllister C.S., Samuel C.E., Safinya C.R. (2010). Cationic liposome-nucleic acid complexes for gene delivery and silencing: Pathways and mechanisms for plasmid DNA and siRNA. Top. Curr. Chem..

[123] Moss K.H., Popova P., Hadrup S.R., Astakhova K., Taskova M. (2019). Lipid Nanoparticles for Delivery of Therapeutic RNA Oligonucleotides. Mol. Pharm..

[124] Sakurai F., Inoue R., Nishino Y., Okuda A., Matsumoto O., Taga T., Yamashita F., Takakura Y., Hashida M. (2000). Effect of DNA/liposome mixing ratio on the physicochemical characteristics, cellular uptake and intracellular trafficking of plasmid DNA/cationic liposome complexes and subsequent gene expression. J. Control. Release.

[125] Dokka S., Toledo D., Shi X., Castranova V., Rojanasakul Y. (2000). Oxygen Radical-Mediated Pulmonary Toxicity Induced by Some Cationic Liposomes. Pharm. Res..

[126] Yan W., Chen W., Huang L. (2008). Reactive oxygen species play a central role in the activity of cationic liposome based cancer vaccine. J. Control. Release.

[127] Knudsen K.B., Northeved H., Kumar P.E., Permin A., Gjetting T., Andresen T.L., Larsen S., Wegener K.M., Lykkesfeldt J., Jantzen K. (2015). In vivo toxicity of cationic micelles and liposomes. Nanomedicine.

[128] Zhanataev A.K., Anisina E.A., Kulakova A.V., Shilovskiy I.P., Lisitsyn A.A., Koloskova O.O., Khaitov M.R., Durnev A.D. (2020). Genotoxicity of cationic lipopeptide nanoparticles. Toxicol. Lett..

[129] Leon-Mejia G., Quintana-Sosa M., de Moya Hernandez Y., Rodriguez I.L., Trindade C., Romero M.A., Luna-Carrascal J., Ortiz L.O., Acosta-Hoyos A., Ruiz-Benitez M. (2020). DNA repair and metabolic gene polymorphisms affect genetic damage due to diesel engine exhaust exposure. Environ. Sci. Pollut. Res..

[130] Liou S.H., Wu W.T., Liao H.Y., Chen C.Y., Tsai C.Y., Jung W.T., Lee H.L. (2017). Global DNA methylation and oxidative stress biomarkers in workers exposed to metal oxide nanoparticles. J. Hazard. Mater..

[131] Rossnerova A., Honkova K., Pelclova D., Zdimal V., Hubacek J.A., Chvojkova I., Vrbova K., Rossner P., Topinka J., Vlckova S. (2020). DNA Methylation Profiles in a Group of Workers Occupationally Exposed to Nanoparticles. Int. J. Mol. Sci..

